# Design of a molecular memory element with an alternating circular array of dipolar rotors and rotation suppressors†

**DOI:** 10.1039/d0sc02836c

**Published:** 2020-07-13

**Authors:** Takuya Miyazaki, Yoshiaki Shoji, Fumitaka Ishiwari, Takashi Kajitani, Takanori Fukushima

**Affiliations:** Laboratory for Chemistry and Life Science, Institute of Innovative Research, Tokyo Institute of Technology 4259 Nagatsuta Midori-ku Yokohama 226-8503 Japan yshoji@res.titech.ac.jp fukushima@res.titech.ac.jp; RIKEN SPring-8 Center 1-1-1 Kouto, Sayo Hyogo 679-5148 Japan; Materials Analysis Division, Open Facility Center, Tokyo Institute of Technology 4259 Nagatsuta, Midori-ku Yokohama 226-8503 Japan; Japan Science and Technology Agency (JST), CREST 4-1-8 Hon-cho Kawaguchi Saitama 332-0012 Japan

## Abstract

As a new element for electric-field driven molecular memory, we developed a hexaarylbenzene derivative in which three difluorophenyl groups and three aryl groups as a dipolar rotor and a rotation suppressor, respectively, are alternately positioned on the central benzene core. This molecule has two rotational isomeric forms, both of which preserve their conformational states at room temperature but exhibit interconversion at high temperatures. Amorphous thin films fabricated from the hexaarylbenzene show a reversible change in surface potential by application of electric fields.

## Abstract 2

A hexaarylbenzene derivative with an alternating circular array of dipolar rotors and rotation suppressors holds promise as a new element for electric-field driven molecular memory.

## Graphical abstract

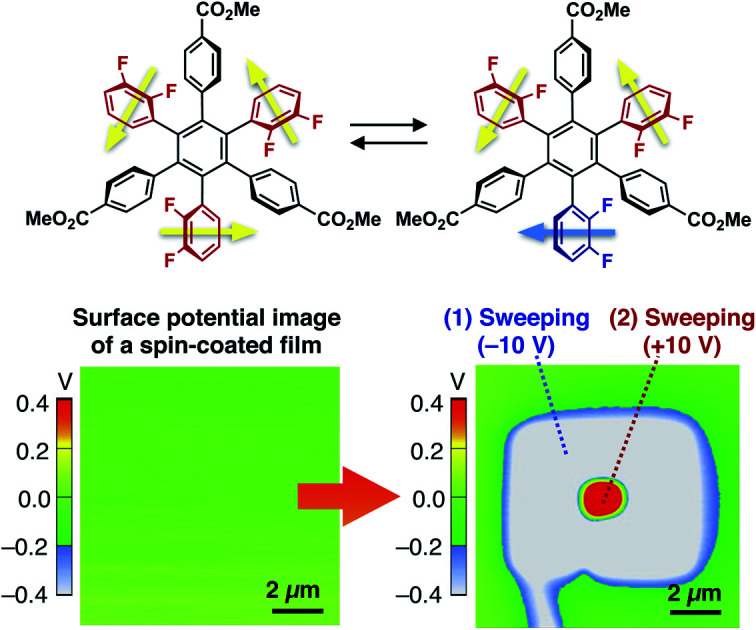

## Introduction

Molecular systems that exhibit changes in structure and physical properties nondestructively and reversibly in response to external stimuli have attracted considerable attention as an element for next-generation nano- and molecular electronics.^1^ One of the ultimate goals in this realm is to realize single-molecule switches and memory operable under ambient conditions by application of electric fields,^2^ in which the molecular elements are required to possess at least two interconvertible stable states (*i.e.*, bistability) with different electronic properties. Ideally, the interconversion of the two states can be achieved in a process without involving redox reactions that generate reactive radical ion species. However, the development of such single-molecule systems is still challenging, and most electric-field-responsive organic systems, as represented by ferroelectric materials, have relied on phase transitions occurring in the bulk solid states.^3^

Single-molecular junctions (SMJs)^4^ and self-assembled monolayers (SAMs)^5^ are promising configurations for achieving electric field-responsive single-molecule switches and memory. Recently, we reported electric-field-controllable conductance switching of overcrowded ethylene-based SMJs and SAMs under ambient conditions.^6,7^ While overcrowded ethylene exhibits folded-to-twisted conformational isomerism similar to common thermochromic ethylenes, it is unique in that the conformational bistability is greatly improved in its SAM state on Au(111) due to the formation of a two-dimensional network by specific intermolecular interactions.^7^ However, a problem with the SMJ and SAM systems is that further chemical modification of the overcrowded ethylene molecule is impossible.

**Fig. 1:**
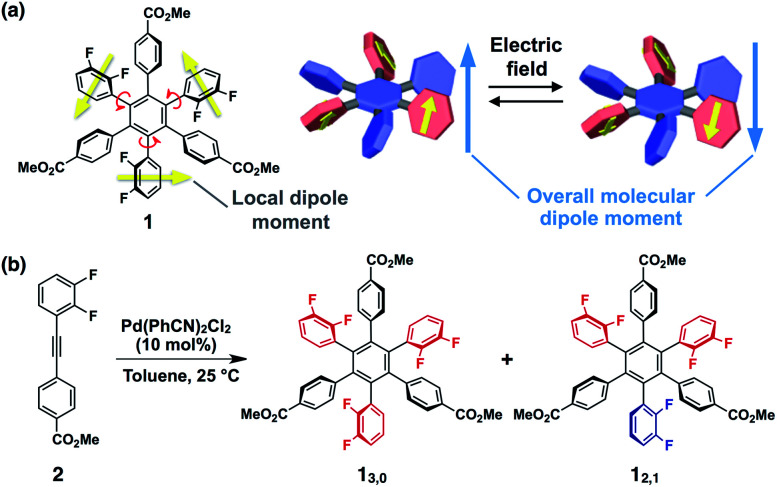
(a) Molecular structure of hexaarylbenzene **1** and schematic illustration of a possible isomerisation with reversal of the dipolar rotor rings. (b) Synthetic scheme of the rotational isomers of **1** (**13,0** and **12,1**).

Here we present a conceptually new element for single molecular switches and memory based on a hexaarylbenzene building block (Fig. 1a). The molecular design concept is based on the idea that the introduction of difluorophenyl groups at the 1,3,5-positions of the central benzene core could lead to not only large dipoles but also a moderate kinetic barrier for ring rotation that results in conformational bistability. This idea was embodied with hexaarylbenzene derivative **1** (Fig. 1a) with an alternating circular array of dipolar rotors (*i.e.*, difluorophenyl rings) and rotation suppressors (*i.e.*, other benzene rings). The ester substituents at the *para*-position of the peripheral benzene groups could be used for post-modifications for monolayer adsorption on metal surfaces or for constructing intermolecular network structures in the solid state. Through the investigation on the conformational behaviour of **1** in solution and in the solid state, we revealed that **1** can give two rotational isomers (**13,0** and **12,1**, Fig. 1b), where in one isomer, the dipoles of all three difluorophenyl rings are arranged in the same direction, while in the other isomer, one of the three difluorophenyl rings is arranged so as to cancel the dipoles of the other two. These two rotational isomers were conformationally stable at room temperature but showed interconversion at high temperatures. We also found that amorphous thin films obtained by spin-coating of a solution of **1** can change the surface potential in response to the sign of applied electric fields, and the resulting surface states were retained for several hours.

## Results and discussion

### Synthesis and conformational behaviour of **1**

**Fig. 2:**
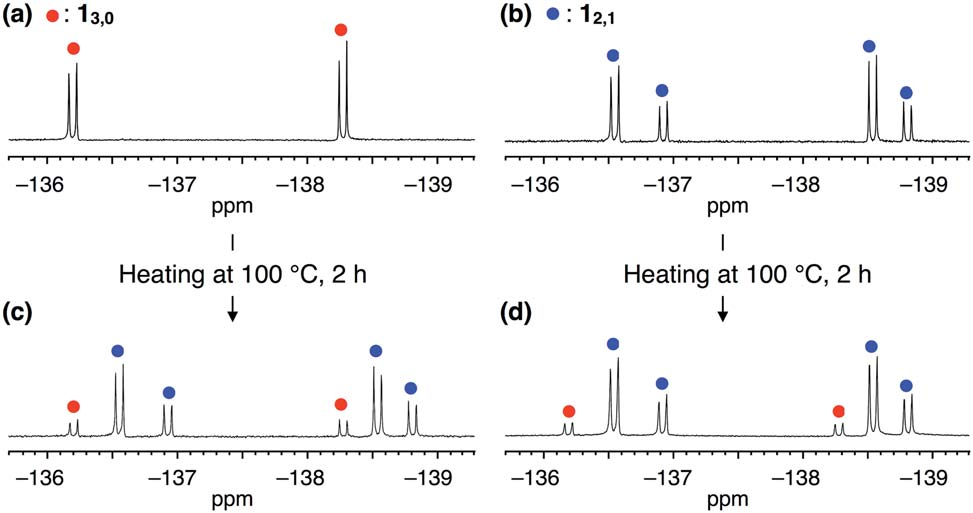
^19^F NMR spectra (376 MHz, 298 K, CDCl_3_) of (a) **13,0**, (b) **12,1** and (c and d) those after heating in toluene at 100 °C for 2 h. Red and blue filled circles represent the signals of **13,0** and **12,1**, respectively.

The synthesis of **1** was achieved by palladium-catalyzed cyclotrimerization^8^ of methyl 4-(2,3-difluorophenylethynyl)benzoate **2** at 25 °C in toluene, affording a mixture of two rotational isomers of **1** (Fig. 1b, ESI†). Since both isomers were conformationally stable in solution at room temperature, high-performance liquid chromatography of the resulting mixture allowed the isolation of **13,0** and **12,1** in 5 and 2% yields, respectively. The structures of these compounds including the configuration of their difluorophenyl rings were successfully identified by ^1^H, ^13^C and ^19^F NMR spectroscopy (ESI†). All three difluorophenyl groups in **13,0** are oriented in the same direction, while in **12,1**, one of the difluorophenyl groups is oriented in the opposite direction to the other difluorophenyl groups (Fig. 1b). Accordingly, the ^19^F NMR spectrum of **13,0** in CDCl_3_ at 298 K displayed a spectral pattern characteristic of *C*_3_-symmetry, where two doublet peaks were observed at −136.2 and −138.3 ppm due to the difluorophenyl groups (Fig. 2a). On the other hand, the ^19^F NMR spectral pattern of **12,1** was lower in symmetry and showed two sets of doublet peaks (−136.5 and −138.5 ppm and −136.9 and −138.8 ppm) with an integral ratio of 2 : 1 (Fig. 2b).

The ^19^F NMR spectra of **13,0** and **12,1** did not change after they were left standing for one week at 298 K. However, at higher temperatures (*e.g.*, at 100 °C in toluene), the interconversion between **13,0** and **12,1** took place, as confirmed by ^19^F NMR spectroscopy (Fig. 2c and d). For example, when a toluene-d_8_ solution of **13,0** was heated at 333 K, the ^19^F NMR signals due to **12,1** gradually appeared, along with the decrease in intensity of those due to **13,0** (Fig. S2, ESI†). After approximately 192 h, the molar ratio of **13,0** : **12,1** reached 1 : 9, which corresponds to the value of the thermal equilibrium state of **1** in solution. Based on the ^19^F NMR spectral change, apparent rate constants for the rotational isomerisations of **13,0** → **12,1** (*k*) and **12,1** → **13,0** (*k*′) at 333 K were determined to be 3.6 × 10^−2^ and 3.8 × 10^−3^ s^−1^, respectively (Fig. S3, ESI†). The *k* and *k*′ values at 343, 353 and 363 K were also determined (Table 1). From the plots of ln(*k*/*T*) or ln(*k*′/*T*) *versus T*^−1^ K^−1^ (Fig. S4, ESI†), thermodynamic parameters (Δ*H*^‡^ and Δ*S*^‡^) for the corresponding isomerisations are obtained as shown in Table 1 (Δ*H*^‡^ = 21.9 ± 1.4 kcal mol^−1^ and Δ*S*^‡^ = 0.5 ± 1.1 cal mol^−1^ K^−1^ for **13,0** → **12,1**, and Δ*H*^‡^ = 23.4 ± 1.4 kcal mol^−1^ and Δ*S*^‡^ = 0.2 ± 4.5 cal mol^−1^ K^−1^ for **12,1** → **13,0**). At 298 K, the activation Gibbs energies (Δ*G*^‡^) for **13,0** → **12,1** and **12,1** → **13,0** were calculated to be 21.7 ± 0.2 and 23.2 ± 0.2 kcal mol^−1^, respectively (Table 1), which are large enough to suppress the rotational isomerisation at room temperature.

**Table 1:** Apparent rate constants and thermodynamic parameters for the rotational isomerisation of **1**

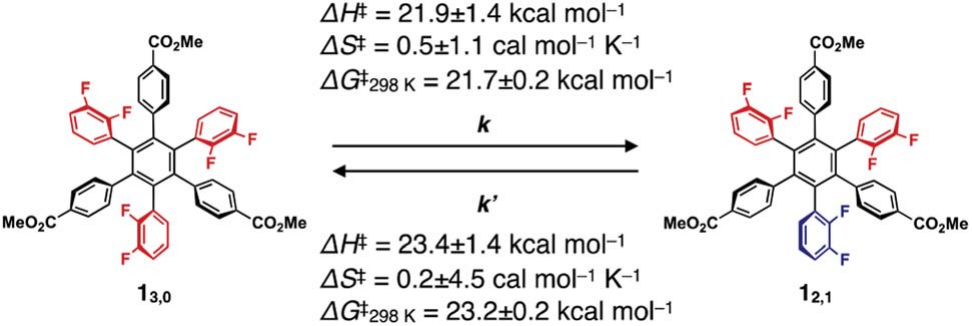				
*T* (K)	333	343	353	363

*k* (s^−1^)	3.6 × 10^−2^	9.1 × 10^−2^	0.21	0.63
*k*′ (s^−1^)	3.8 × 10^−3^	9.9 × 10^−3^	2.4 × 10^−2^	8.0 × 10^−2^

**Fig. 3:**
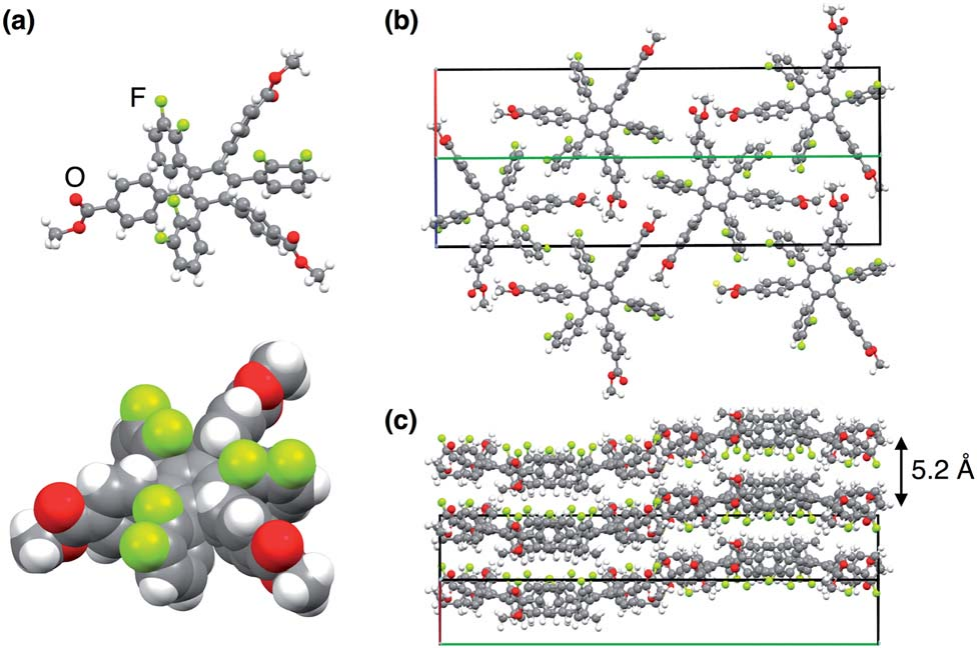
(a) X-ray structure of **13,0** (top; ball and stick and bottom; CPK descriptions) and (b and c) the packing diagrams. In (b and c), red, green and blue lines represent *a*, *b* and *c* axes of the unit cell, respectively.

Upon recrystallisation from a mixture of CHCl_3_ and hexane at 25 °C, **13,0** and **12,1** afforded high-quality crystals suitable for X-ray diffraction analysis (Fig. 3 and S1, ESI†). The X-ray crystal structures of **13,0** and **12,1** both showed the formation of quasi two-dimensional sheets in which the aryl groups interpenetrate intermolecularly and are arranged parallel to one another, resulting in a layered structure (Fig. 3b, c, S1b and c, ESI†). Although this interpenetration appeared to suppress ring rotation in the solid state, we observed that the interconversion between **13,0** and **12,1** proceeds partly at a high temperature (*e.g.*, at 240 °C).

Crystalline samples of **13,0** and **12,1** did not melt below 250 °C under the optical microscope, and thermogravimetric analysis (TGA) indicated that the weight loss of **1** is only 1% even when heated to *ca.* 300 °C (Fig. S5, ESI†). In differential scanning calorimetry (DSC), while a crystalline sample of **12,1** did not show any clear phase transition feature in a temperature range of 25–250 °C, that of **13,0** displayed a small endothermic peak (−5.3 kJ mol^−1^) at 88.3 °C in the first heating run, which is due to crystal-to-crystal transition (Fig. S6, ESI†). The corresponding endothermic peak appeared at lower temperatures in the second (79.0 °C) and third (74.2 °C) heating runs, suggesting the occurrence of some type of structural changes in the molecular and/or assembly structure upon heating. Indeed, when a crystalline sample of **13,0** was heated to 240 °C, a mixture of **13,0** and **12,1** gradually formed (Fig. S7, ESI†) but did not reach a thermal equilibrium even after prolonged heating for 6 h.

### Thin-film fabrication

**Fig. 4:**
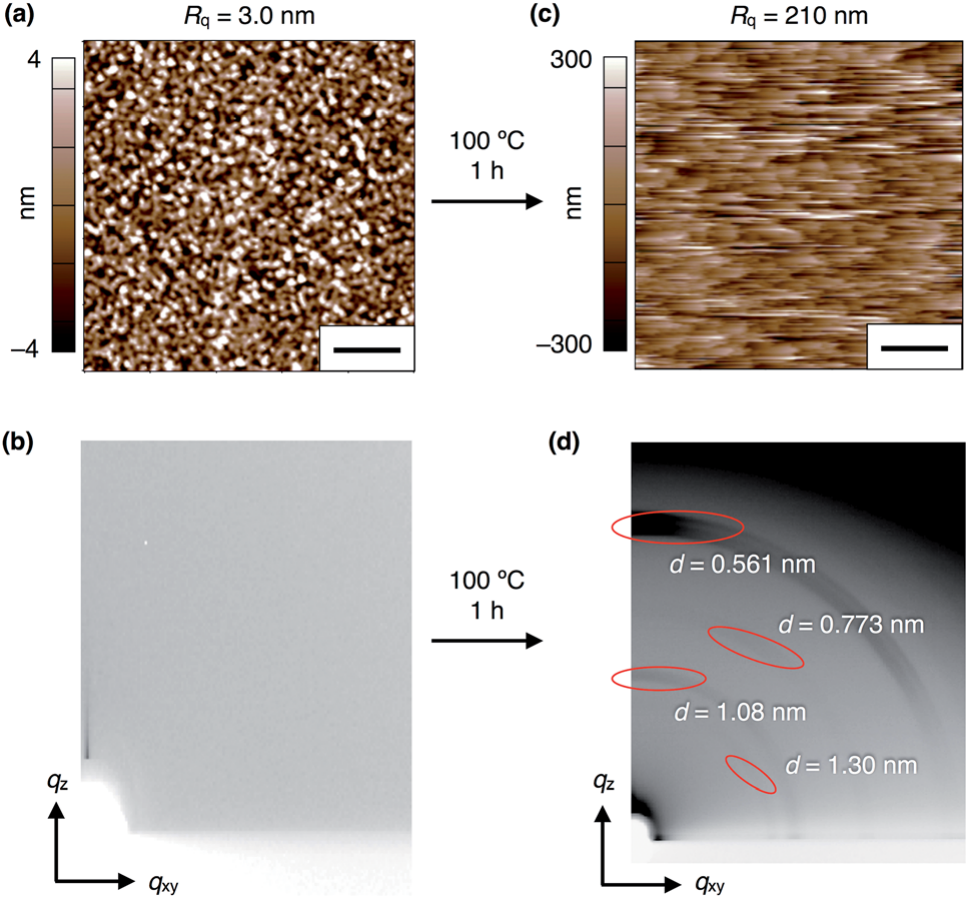
(a) AFM and (b) 2D GI-XRD images of a 40 nm-thick spin-coated amorphous film of **13,0** on ITO. (c) AFM and (d) 2D GI-XRD images of a thermally crystallized film of **13,0** on ITO. Scale bars = 2 μm.

To investigate the electric-field response of **1**, we prepared thin-film samples on an indium tin oxide (ITO) substrate (1 cm × 2 cm) by spin-coating (1000 rpm) a CHCl_3_ solution (10 mg mL^−1^) of **1**. Atomic force microscopy (AFM) imaging of a 40 nm-thick film of **13,0** visualized a flat surface of the film (Fig. 4a), where a root-mean-square deviation (*R*_q_) in the measurement area was 3.0 nm. In the as-prepared films, **13,0** was found to adopt an amorphous state, since no detectable diffraction was observed in grazing-incidence X-ray diffraction (GI-XRD) experiments using a synchrotron X-ray beam (*λ* = 1.0 Å) (Fig. 4b). In contrast, a spin-coated film of **13,0**, after being thermally annealed at 100 °C for 1 h, clearly displayed oriented diffraction arcs in its 2D GI-XRD image (Fig. 4d). The 1D diffraction pattern obtained by converting the 2D GI-XRD data was similar to that simulated from the single-crystal X-ray data of **13,0** (Fig. 3, 4d and S8, ESI†). Thus, thermal annealing triggers structuring of amorphous **13,0** into a crystal state with a structure similar to that formed in its single crystal. Given the fact that the diffraction arc with a *d*-spacing of 0.561 nm was observed in the meridional direction in the 2D GI-XRD image, a layer structure is formed vertical to the substrate. The AFM image of a thermally annealed film of **13,0** showed a significant increase in surface roughness (*R*_q_ = 210 nm) due to the formation of sub μm-sized crystalline domains (Fig. 4c). As in the case of **13,0**, spin-coating of a CHCl_3_ solution of **12,1** on an ITO substrate gave rise to an amorphous thin film, which turned crystalline upon thermal annealing at 100 °C (Fig. S9 and S10, ESI†).

### Electric-field response of the thin films of **1**

Notably, we found that the as-spin-coated amorphous thin films of both **13,0** and **12,1** exhibit an electric-field responsive behaviour, where positive and negative surface potentials were induced and memorized upon application of electric fields. In contrast, the corresponding crystalline films of **13,0** and **12,1**, obtained after thermal annealing, did not show such a change in surface potential under identical conditions (Fig. S11 and S12, ESI†). This is most likely due to interpenetrated molecular packing of the aryl groups, which eliminates a free volume for ring rotation.

**Fig. 5:**
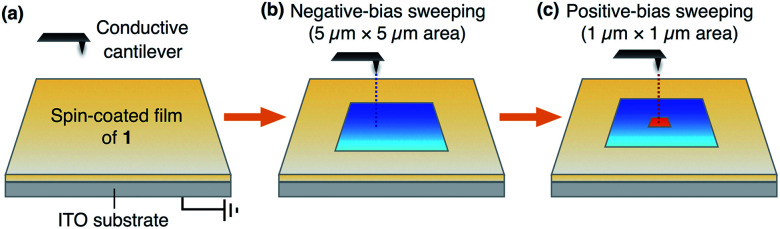
Schematic illustration of the experimental setup for conductive AFM measurements to apply electric fields to a thin film of **1** on ITO.

Fig. 5 shows a schematic illustration of the experimental setup and procedures for applying an electric field to a thin film of **1** using a conductive AFM system equipped with a platinum-coated silicon cantilever (ESI†). Positive and negative electric fields were applied to a thin film by contact-mode electrical force microscopy (EFM).^9^ In the first scan, a bias voltage of −10 V was applied to a square region (5 μm × 5 μm) of the film surface, and then, a bias voltage with an opposite sign (+10 V) was applied to an inner smaller region (1 μm × 1 μm). Before and after the application of electric fields, the surface potential on the film was measured by scanning Kelvin probe force microscopy (SKPM) with an alternating current (AC)-mode,^10^ where the mean surface potential of the intact film was used as a standard (0 V).

**Fig. 6:**
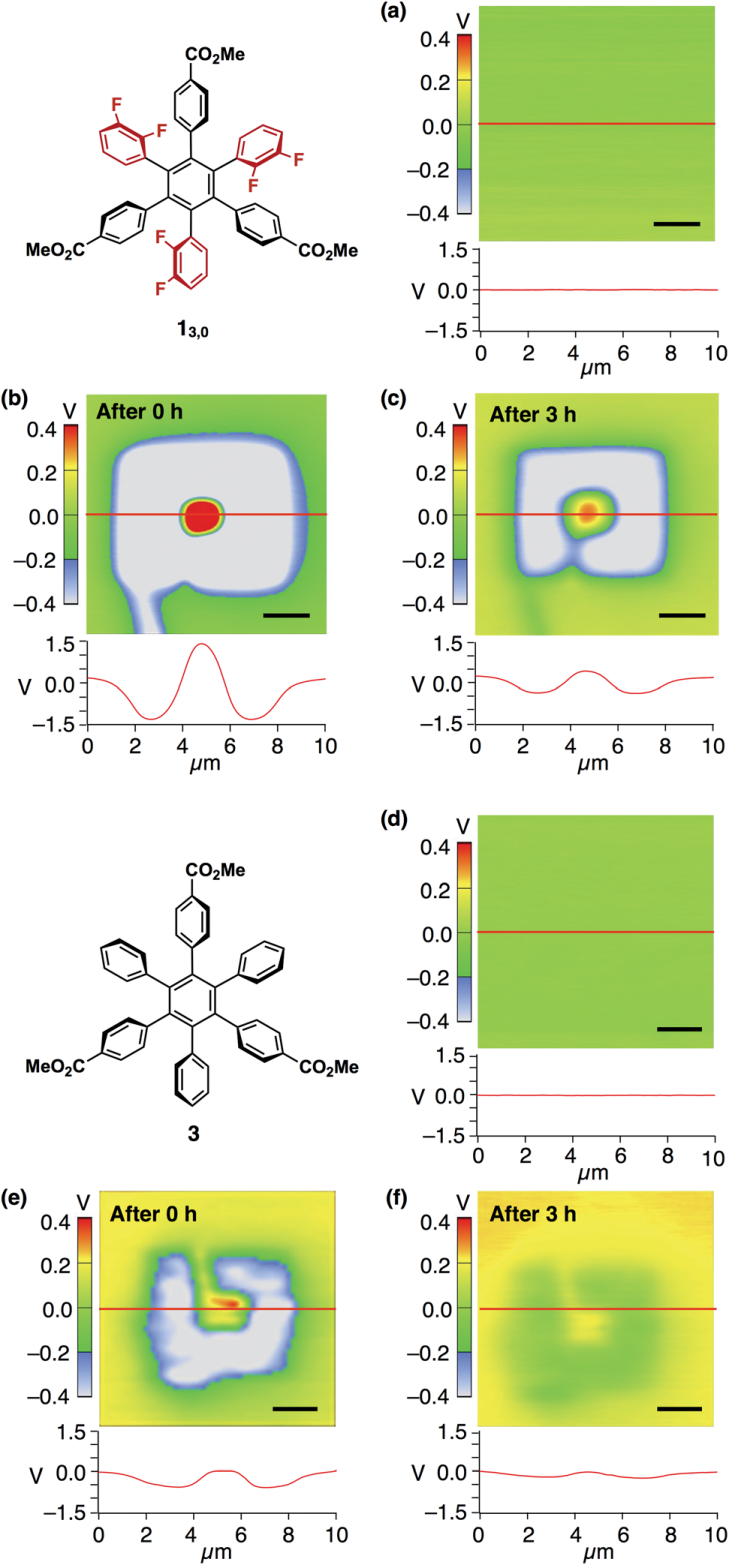
(a) SKPM image (top) and surface potential trace (bottom) of a 40 nm-thick spin-coated amorphous film of **13,0** on ITO and those measured (b) just after the application of bias voltages of ±10 V and (c) 3 h later. (d) SKPM image (top) and surface potential traces (bottom) of a 40 nm-thick spin-coated amorphous film of **3** on ITO and those measured (e) just after the application of bias voltages of ±10 V and (f) 3 h later. Bias voltages were applied according to the procedure illustrated in Fig. 5. The surface potential traces were obtained by scanning along the red line. Scale bars = 2 μm. The corresponding AFM image of the films of **13,0** and **3** are shown in Fig. 4a and S14,† respectively.

**Fig. 7:**
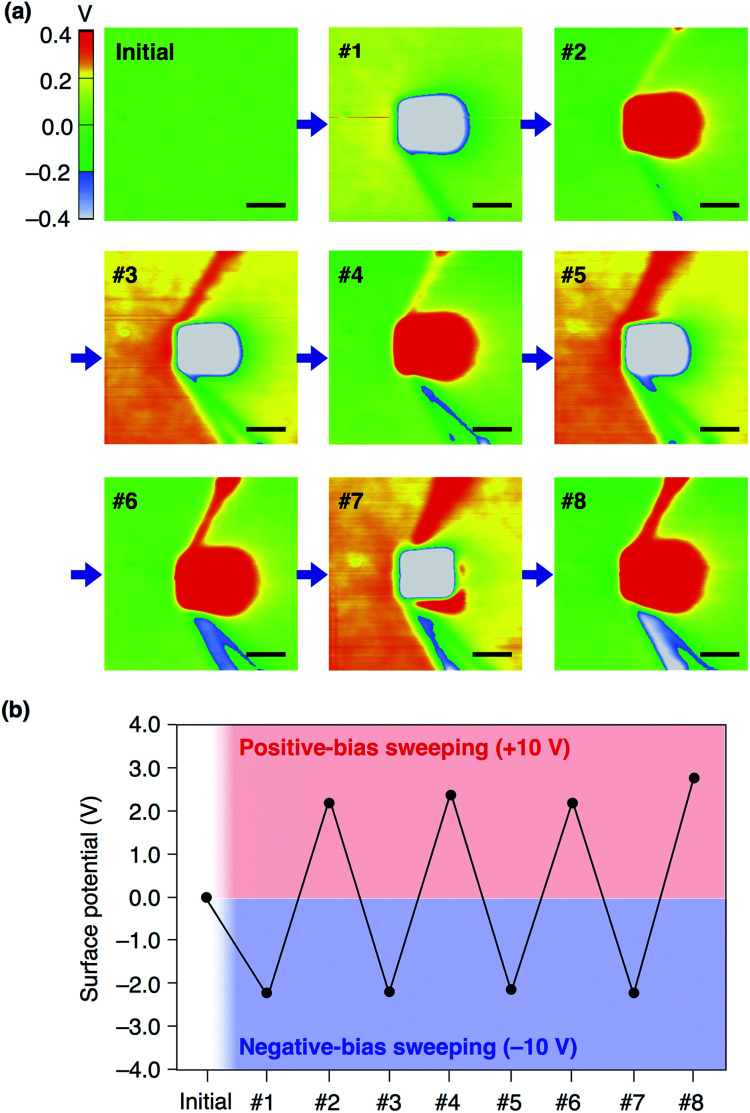
(a) SKPM images of a 40 nm-thick spin-coated amorphous film of **13,0** on ITO measured before (initial) and after (#1–#8) alternately applying negative and positive bias voltages (−10 and +10 V) at the central region (1 μm × 1 μm). Scale bars = 1 μm. (b) Plots of induced surface potential values in (a).

We confirmed that surface potential of an amorphous film of **13,0** is initially uniform (Fig. 6a). When an electric field was applied, negative and positive surface potentials were observed in the outer (5 μm × 5 μm) and inner (1 μm × 1 μm) regions, respectively (Fig. 6b). The signs of the induced surface potential agreed with those of the applied bias voltages. This indicates that the negative and positive surface potential of the film can be reversibly induced by the application of electric fields. Importantly, the induced surface potential was maintained for at least 3 h (Fig. 6c), albeit decreasing in its magnitude over time. The half-life times for the induced negative and positive surface potentials were determined to be 1.33 ± 0.14 and 0.73 ± 0.11 h, respectively (Fig. S13, ESI†). Therefore, electrically induced biased states of the film have substantial stability at room temperature. Furthermore, when the negative and positive voltages were alternately applied to the same surface area of an amorphous film of **13,0**, the corresponding negative and positive surface potentials were induced reproducibly (Fig. 7a and b). An amorphous film of **12,1** showed electric field-responsive behaviour similar to that observed for the amorphous film of **13,0** in terms of the sign of the induced surface potential (Fig. S12a–c, ESI†).

The fact that the signs of the induced surface potentials are identical to those of the applied electric field (Fig. 5, 6b and c) is different from the case of usual ferroelectric materials, where the dipole moments of constituent building blocks align in a direction so as to cancel an applied electric field.^11^ At present, the mechanism at the molecular level for the observed surface potential modulation is unclear, we presume that a screen effect,^12^ which has been often reported for thin films of dielectric materials,^13^ might be responsible for determining the sign of the induced surface potential. This effect is a phenomenon in which, when an electric field is applied, water molecules and/or charged particles in air adsorb to the surface of a material,^14^ and the apparent surface potential of the material weakens or reverses in response to the applied electric field.

Nonetheless, we would like to emphasize here that the dipolar rotating units of **1** play a key role in the observed electric-field response. This was verified by a control experiment using an amorphous film prepared from **3** (Fig. 6d and S14, ESI†). This compound is very similar to **1** in structure but lacks fluorine atoms. Upon application of electric fields, positive and negative surface potentials on this film were induced weakly, while they disappeared much more rapidly compared to the case of the amorphous films of **1** (Fig. 6e and f). Indeed, the half-life time for the induced negative surface potential was 0.97 ± 0.06 h and that for the induced positive surface potential was too short to evaluate (Fig. S13, ESI†). This result also indicates that the dipolar rotating units rather than the ester groups contribute to the observed electric field-response.

The amorphous **13,0** film can respond to bias voltages less than ±10 V. Fig. S15 shows surface potential images of an amorphous film of **13,0** after applying electric fields of ±5, ±3 and ±1 V (ESI).† We observed an almost comparable intensity of the induced positive surface potential (*ca*. 0.9 V) irrespective of the magnitude of the applied positive bias voltage. In contrast, the intensity of the induced negative surface potential was largely dependent on the magnitude of the applied negative bias voltage. These observations demonstrate the possibility of being able to make patterning of the surface-potential distribution on the film of **1**. We believe that the interesting bias voltage dependence could be clarified by experiments using a self-assembled monolayer of **1**, which is our next target in this molecular rotor system.

## Conclusions

In conclusion, we have shown the molecular design concept, conformational behaviours and electric field-responsiveness of a dipolar rotor-containing a hexaarylbenzene derivative (**1**) that serves as a new building block for molecular memory. This compound has thermally interconvertible two rotational isomers, which are conformationally stable at room temperature both in solution and in the solid state. Amorphous thin films fabricated with **1** were found to exhibit reversible switching of the surface potential in response to applied bias voltages, where the induced biased states have substantial stability at room temperature. These observations prove our design concept of **1** that the rotatable dipolar units, when arranged circularly to cause a moderate steric hindrance by the neighboring phenyl rings, result in an electric field-responsive property. We are continuing research that focuses on constructing a new single molecular switch and memory systems using self-assembled monolayers of derivatives of **1** with suitable chemical modifications at the 4-positions of the phenylene rings.

## Conflicts of interest

There are no conflicts to declare.

## Supplementary Material

SC-011-D0SC02836C-s001

SC-011-D0SC02836C-s002
