# Supplementary material for: Design of a molecular memory element with an alternating circular array of dipolar rotors and rotation suppressors
Source: Chem Sci. 2020 Jul 13;11(32):8388–93. doi: 10.1039/d0sc02836c (PMC8163413; doi:10.1039/d0sc02836c)
Supplement: SC-011-D0SC02836C-s001 [file SC-011-D0SC02836C-s001.pdf]

Electronic Supplementary Material (ESI) for Chemical Science.
This journal is © The Royal Society of Chemistry 2020

Supplementary Information

Design of a molecular memory element with an alternating circular array of dipolar rotors and rotation suppressors

Takuya Miyazaki, Yoshiaki Shoji,* Fumitaka Ishiwari, Takashi Kajitani and Takanori Fukushima*

*To whom correspondence should be addressed.
E-mail: yshoji@res.titech.ac.jp (Y.S.), fukushima@res.titech.ac.jp (T.F.)

Table of Contents

1. **Materials** S2
2. **Methods** S2
3. **Synthesis** S2
4. **Synchrotron-Radiation X-ray Scattering Experiments** S4
5. **Single-Crystal X-Ray Crystallography** S4
6. **Supplementary Figures (Figs. S1–S15)** S5
7. **Supplementary References** S14
8. **Analytical Data (Figs. S16–S22)** S15

1. Materials

Unless otherwise noted, all commercial reagents were used as received. ITO substrates (10 Ω/□) were purchased from EHC Co., Ltd. 1,3,5-tris(4-methoxycarbonylphenyl)-2,4,6-triphenylbenzene[1] (**3**) was prepared according to the previously reported procedure. Anhydrous toluene was dried by passage through an activated alumina column and a Q-5 column (Nikko Hansen & Co., Ltd.).

2. Methods

Air- and/or moisture-sensitive compounds were handled by applying a standard Schlenk-line technique. Column chromatography was carried out using Silica Gel 60N (particle size: 63–210 μm). High performance liquid column chromatography (HPLC) was carried out on a Japan Analytical Industry model LC-9210 NEXT recycling preparative HPLC system equipped with a Mightysil column (20 mm × 250 mm, Kanto Chemical Industry Co., Ltd.). Infrared (IR) spectra were recorded at 25 °C on a JASCO model FT/IR-660*Plus* Fourier transform IR spectrometer. Nuclear magnetic resonance (NMR) spectroscopy measurements were carried out on a Bruker model AVANCE-400 spectrometer (^{1}H: 400.0 MHz, ^{13}C: 100.6 MHz, ^{19}F: 376.3 MHz) or on a Bruker model AVANCE III HD-500 spectrometer (^{1}H: 500.0 MHz, ^{13}C: 125.7 MHz, ^{19}F: 470.4 MHz). Chemical shifts (δ) are expressed relative to the resonances of the residual non-deuterated solvent for ^{1}H ($CDCl_3$: ^{1}H(δ) = 7.26 ppm, toluene-d_8: ^{1}H(δ) = 7.09, 7.01, 6.97 and 2.08 ppm), the resonances of the residual solvent for ^{13}C ($CDCl_3$: ^{13}C(δ) = 77.16 ppm, toluene-d_8: ^{13}C(δ) = 137.48, 128.87, 127.96, 125.13 and 20.43 ppm), the resonance of CF_3COOH as external standard for ^{19}F (CF_3COOH: ^{19}F(δ) = –76.55 ppm). Absolute values of the coupling constants are given in Hertz (Hz), regardless of their sign. Multiplicities are abbreviated as singlet (s), doublet (d), triplet (t), quartet (q), multiplet (m) and broad (br). Mass spectrometry measurements were carried out on a Bruker model micrOTOF II mass spectrometer equipped with an atmospheric pressure chemical ionization (APCI) probe. Thermogravimetric analysis was performed on a Shimadzu model TGA-50 thermogravimetric analyzer. Differential scanning calorimetry was carried out on a Mettler-Toledo model DSC-1 differential scanning calorimeter, where the heating profile was recorded and analyzed using a Mettler-Toledo STAR^e software system. Spin-coating was performed on a MIKASA model 1H-D7 spin-coater. Atomic force microscopy (AFM) and scanning Kelvin probe force microscopy (SKPM) was performed on an Oxford Instruments model Cypher atomic force microscope, where all the measurements were conducted in air at 25 °C. While a tapping mode was employed for AFM and SKPM measurements, a contact mode was used for applying bias voltages. A silicon cantilever (Olympus, OMCL-AC160TS-R3) and a platinum-coated silicon cantilever (Olympus, OMCL-AC240TM-B3) were used for the AFM and SKPM measurements, respectively.

3. Synthesis

Compound 2. Under argon, 1-bromo-2,3-difluorobenzene (1.60 mL, 14.1 mmol) was added to a Et_3N suspension (40 mL) of a mixture of methyl 4-ethynylbenzoate (1.50 g, 9.37 mmol), $Pd(PPh_3)_2Cl_2$ (329 mg, 0.468 mmol) and CuI (178 mg, 4.65 mmol), and the reaction mixture was stirred at 60 °C for 38 h. After allowed to cool to 25 °C, the resulting mixture was diluted with Et_2O (50 mL), passed through a plug of Celite® and evaporated under a reduced pressure. The residue was dissolved in CH_2Cl_2/hexane (v/v = 1/1),

passed through a plug of silica gel, and then recrystallized from $CHCl_3$/hexane to give **2** (0.795 g, 4.97 mmol) as colorless crystals in 53% yield: FT-IR (KBr): ν (cm^{-1}) 3035, 3008, 2959, 1717, 1607, 1602, 1597, 1482, 1471, 1437, 1404, 1310, 1278, 1284, 1196, 1170, 1107, 1018, 979, 857, 786, 770, 716, 693. ^{1}H NMR (400 MHz, $CDCl_3$, 25 °C) δ (ppm): 8.04 (d, J = 8.5 Hz, 2H), 7.62 (d, J = 8.5 Hz, 2H), 7.31-7.26 (m, 1H), 7.22–7.14 (m, 1H), 7.11–7.05 (m, 1H), 3.94 (s, 3H). ^{13}C NMR (125 MHz, $CDCl_3$, 25 °C): δ (ppm) 166.4, 151.0 (d, $^{1}J_{C–F}$ = 254 Hz), 150.6 (d, $^{1}J_{C–F}$ = 254 Hz), 131.7, 130.1, 129.6, 128.3, 127.1, 124.1, 118.0, 113.7, 94.6, 84.2, 52.3. ^{19}F NMR (376 MHz, $CDCl_3$, 25 °C): δ (ppm) –135.6 (d, J = 20 Hz), –138.2 (d, J = 20 Hz). High-resolution APCI-TOF mass: calcd. for $C_{16}H_{10}O_2F_2$ $[M]^+$: m/z = 272.0643; found: 272.0629. ^{1}H, ^{13}C and ^{19}F NMR spectra of **2** are shown in Figs. S16, S17 and S18, respectively.

Compounds $\mathbf{1_{3,0}}$ and $\mathbf{1_{2,1}}$. Under argon, a toluene solution (10 mL) of **2** (1.00 g, 3.67 mmol) and $Pd(PhCN)_2Cl_2$ (141 mg, 0.367 mmol) was stirred at 25 °C for 2 days. The resulting mixture was passed through a plug of Celite® and evaporated under a reduced pressure. The reside was subjected column chromatography on SiO_2 (AcOEt/hexane; v/v = 1/2) to afford yellow solid containing a mixture of cyclotrimerized products. The mixture was subjected to HPLC (Mightysil Si60 column, CH_2Cl_2/hexane; v/v = 9/1) to allow isolation of $\mathbf{1_{3,0}}$ (50 mg, 0.061 mmol) and $\mathbf{1_{2,1}}$ (20 mg, 0.024 mmol) as colorless crystalline materials in 5 and 2% yield, respectively.

$\mathbf{1_{3,0}}$: FT-IR (KBr): ν (cm^{-1}) 3051, 3006, 2950, 2919, 2851, 1730, 1617, 1612, 1589, 1485, 1475, 1438, 1401, 1271, 1113, 983, 770, 731. ^{1}H NMR (400 MHz, $CDCl_3$, 25 °C): δ (ppm) 7.69 (dd, J = 8.0, 1.6 Hz, 3H), 7.61 (dd, J = 8.0, 1.6 Hz, 3H), 7.18 (d, J = 8.1 Hz, 3H), 6.94 (dd, J = 8.1, 1.6 Hz, 3H), 6.76–6.68 (m, 3H), 6.65–6.58 (m, 6H), 3.81 (s, 9H). ^{13}C NMR (125 MHz, $CDCl_3$, 25 °C): δ (ppm) 166.7, 150.1 (dd, $^{1}J_{C–F}$ = 248 Hz), 150.6 (dd, $^{1}J_{C–F}$ = 248 Hz), 143.4, 141.2, 133.4, 129.8, 129.4, 129.0, 128.9, 128.5, 128.0, 126.9, 122.9, 116.5, 116.3, 52.0. ^{19}F NMR (376 MHz, $CDCl_3$, 25 °C): δ (ppm) –136.2 (d, J = 22 Hz), –138.2 (d, J = 22 Hz). High-resolution APCI-TOF mass: calcd. for $C_{48}H_{30}O_6F_6$ $[M]^+$: m/z = 816.1941; found: 816.1936. ^{1}H, ^{13}C and ^{19}F NMR spectra of $\mathbf{1_{3,0}}$ are shown in Figs. S19, S20 and 2a (main text), respectively.

$\mathbf{1_{2,1}}$: FT-IR (KBr): ν (cm^{-1}) 3048, 2994, 2954, 2850, 1728, 1624, 1610, 1591, 1485, 1475, 1435, 1403, 1276, 1213, 1178, 1115, 1020, 984, 861, 775, 770, 730, 710. ^{1}H NMR (400 MHz, $CDCl_3$, 25 °C): δ (ppm) 7.70 (dd, J = 8.0, 1.6 Hz, 1H), 7.65 (dd, J = 6.8, 2.2 Hz, 4H), 7.61 (dd, J = 8.0, 1.6 Hz, 1H), 7.19 (d, J = 8.1 Hz, 1H), 7.07 (d, J = 8.6 Hz, 4H), 6.96 (dd, J = 8.1, 1.6 Hz, 1H), 6.76–6.68 (m, 3H), 6.67–6.60 (m, 6H), 3.81 (s, 9H). ^{13}C NMR (125 MHz, $CDCl_3$, 25 °C): δ (ppm) 166.7, 150.1 (dm, $^{1}J_{C–F}$ = 248 Hz), 147.4 (dm, $^{1}J_{C–F}$ = 248 Hz), 143.4, 142.0, 133.4, 129.8, 129.5, 129.4, 129.2, 128.9, 128.6, 128.5, 128.1, 126.9, 123.1, 116.4, 116.3, 52.0. ^{19}F NMR (376 MHz, $CDCl_3$, 25 °C): δ (ppm) –136.5 (d, J = 22 Hz), –136.9 (d, J = 22 Hz), –138.5 (d, J = 22 Hz), –138.8 (d, J = 22 Hz). High-resolution APCI-TOF Mass: calcd. For $C_{48}H_{30}O_6F_6$ $[M]^+$: m/z = 816.1941; found: 816.1926. ^{1}H, ^{13}C and ^{19}F NMR spectra of $\mathbf{1_{2,1}}$ are shown in Figs. S21, S22 and 2b (main text), respectively.

4. Synchrotron-Radiation X-ray Scattering Experiments.

The grazing incidence X-ray diffraction (GI-XRD) images were obtained using the BL45XU beamline at SPring-8 (Hyogo, Japan) equipped with a Pilatus3X 2M (Dectris) detector. The scattering vector, $q = 4\pi\sin\theta/\lambda$, and the position of the incident X-ray beam on the detector were calibrated using several orders of layer reflections from silver behenate (d = 58.380 Å), where 2θ and λ refer to the scattering angle and wavelength of the X-ray beam (1.0 Å), respectively. The sample-to-detector distance was 0.3 m. The obtained diffraction images were integrated along the Debye–Scherrer ring to afford 1D intensity data using the FIT2D[2] software.

5. Single-Crystal X-ray Crystallography

Colourless pillar and block single crystals of $\mathbf{1}_{3,0}$ and $\mathbf{1}_{2,1}$, respectively, obtained by recrystallization from a mixture of $CHCl_3$ and hexane at 25 °C, was coated with immersion oil (type B: Code 1248, Cargille Laboratories, Inc.) and mounted on a MicroMount (MiTeGen, LLC.). Diffraction data were collected at 90 K under a cold nitrogen gas stream on a Bruker model APEX2 platform-CCD X-ray diffractometer system, using graphite-monochromated Mo-Kα radiation (λ = 0.71073 Å). Intensity data were collected by an ω-scan with 0.5° oscillations for each frame. Bragg spots were integrated using the ApexII program package,[3] and the empirical absorption corrections (multi-scan) were applied using the SADABS program.[4] Structure was solved by a direct method (SHELXT Version 2014/4)[5] and refined by full-matrix least squares (SHELXL Version 2014/5).[6] Anisotropic temperature factors were applied to all non-hydrogen atoms. Hydrogen atoms were placed at calculated positions and refined by applying riding models.

Crystal data for $\mathbf{1}_{3,0}$: colourless block, 0.32 x 0.02 x 0.10 mm^3, Monoclinic, $P2_1/n$, a = 9.423(2) Å, b = 40.326(8) Å, c = 10.692(2) Å, β = 107.30(3)°, V = 3879.1(15) Å^3, Z = 4, density$_{calcd}$ = 1.398 g cm^{-3}, T = 90 K, $2\theta_{max}$ = 48.5°, Mo$K\alpha$ radiation, λ = 0.71073 Å, μ = 0.111 mm^{-1}, 21971 reflections measured, 6258 unique reflections, 544 parameters, GOF = 0.973, $R1$ = 0.0522 ($I > 2\sigma(I)$), w$R2$ = 0.1219 (all data), $\Delta\rho_{min, max}$ = –0.276, 0.246 e Å^{-3}, CCDC-2004173.

Crystal data for $\mathbf{1}_{2,1}$: colourless block, 0.45 x 0.38 x 0.35 mm^3, Triclinic, $P\bar{1}$, a = 11.3765(16) Å, b = 13.951(2) Å, c = 14.046(2) Å, α = 69.423(2)°, β = 70.729(2)°, γ = 86.627(2)°, V = 1965.9(5) Å^3, Z = 2, density$_{calcd}$ = 1.380 g/cm^3 g cm^{-3}, T = 90 K, $2\theta_{max}$ = 58.2°, Mo$K\alpha$ radiation, λ = 0.71073 Å, μ = 0.109 mm^{-1}, 17984 reflections measured, 10450 unique reflections, 801 parameters, GOF = 1.088, $R1$ = 0.0558 ($I > 2\sigma(I)$), w$R2$ = 0.1467 (all data), $\Delta\rho_{min, max}$ = –0.386, 0.877 e Å^{-3}, CCDC-2004174.

6. Supplementary Figures

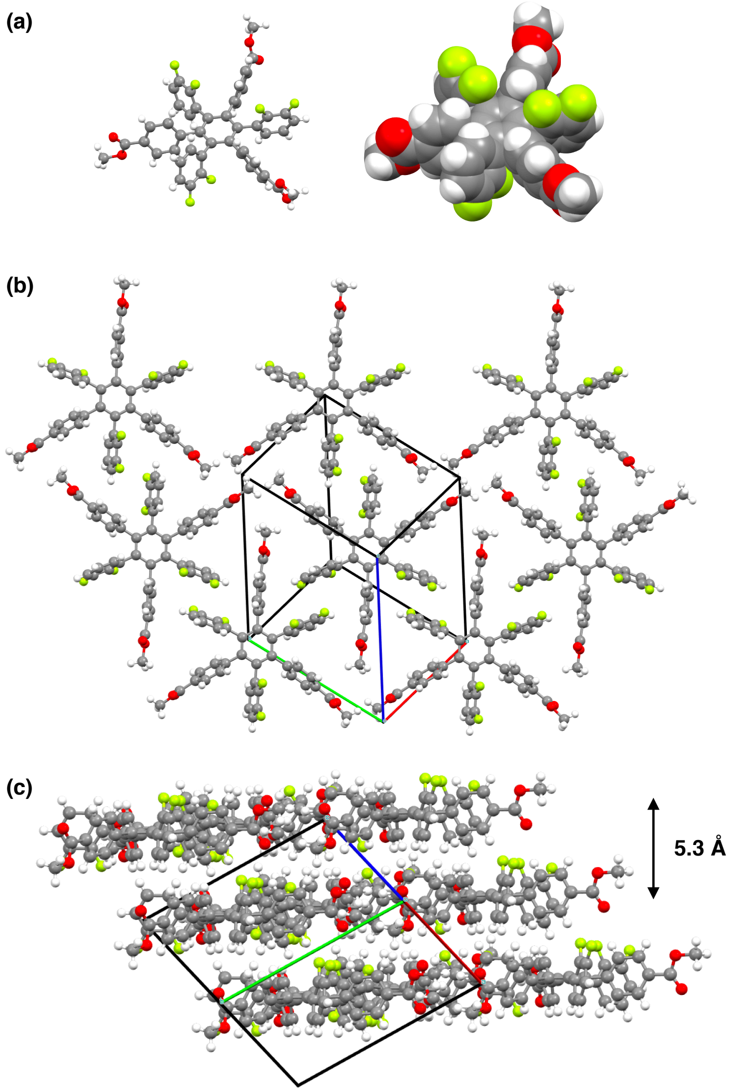

Fig. S1. (a) X-ray structure of $\mathbf{1}_{2,1}$ (left; ball and stick and right; CPK descriptions) and (b,c) the packing diagrams. In (b,c), red, green and blue lines represent *a*, *b* and *c* axes of the unit cell, respectively.

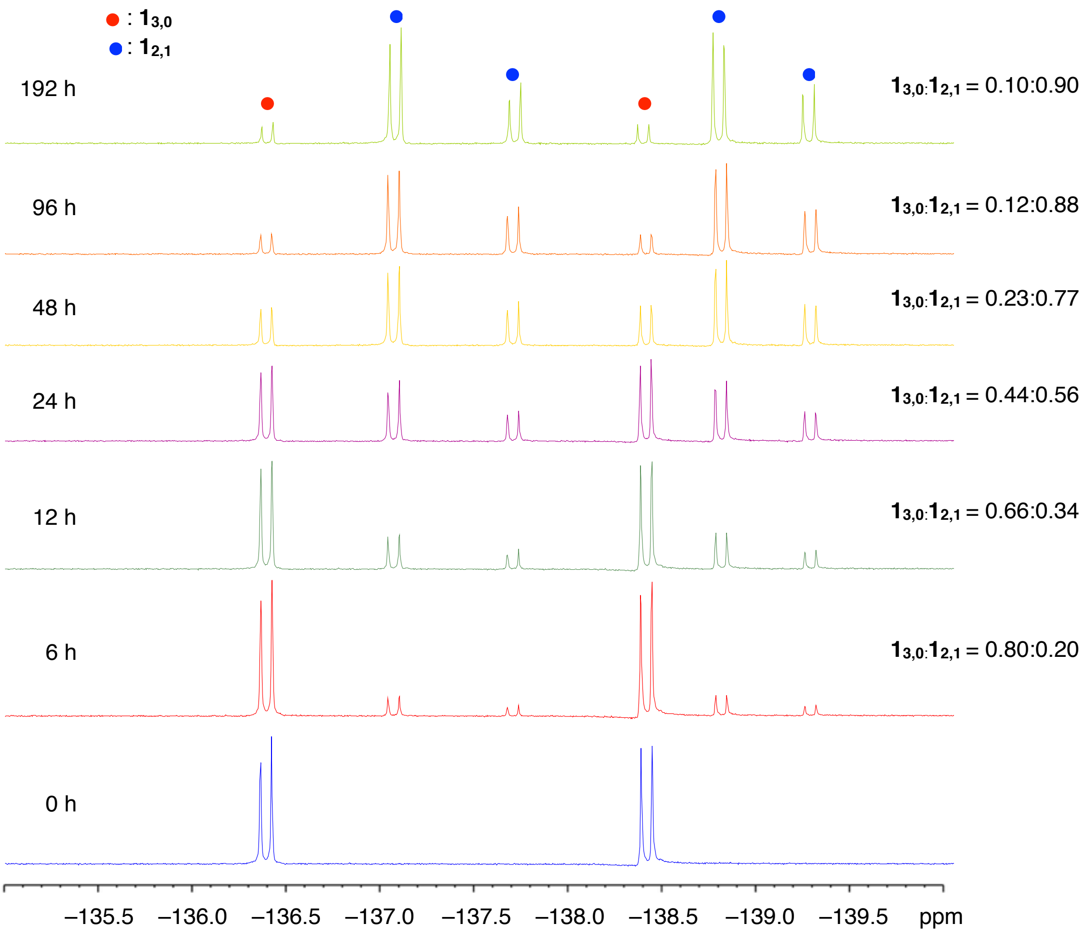

Fig. S2. ^{19}F NMR spectra (376 MHz) of $\mathbf{1}_{3,0}$ at 25 °C in toluene-d_8 after being heated at 333 K for 0–192 h. Initial concentration [$\mathbf{1}_{3,0}$] = 6.7 mM.

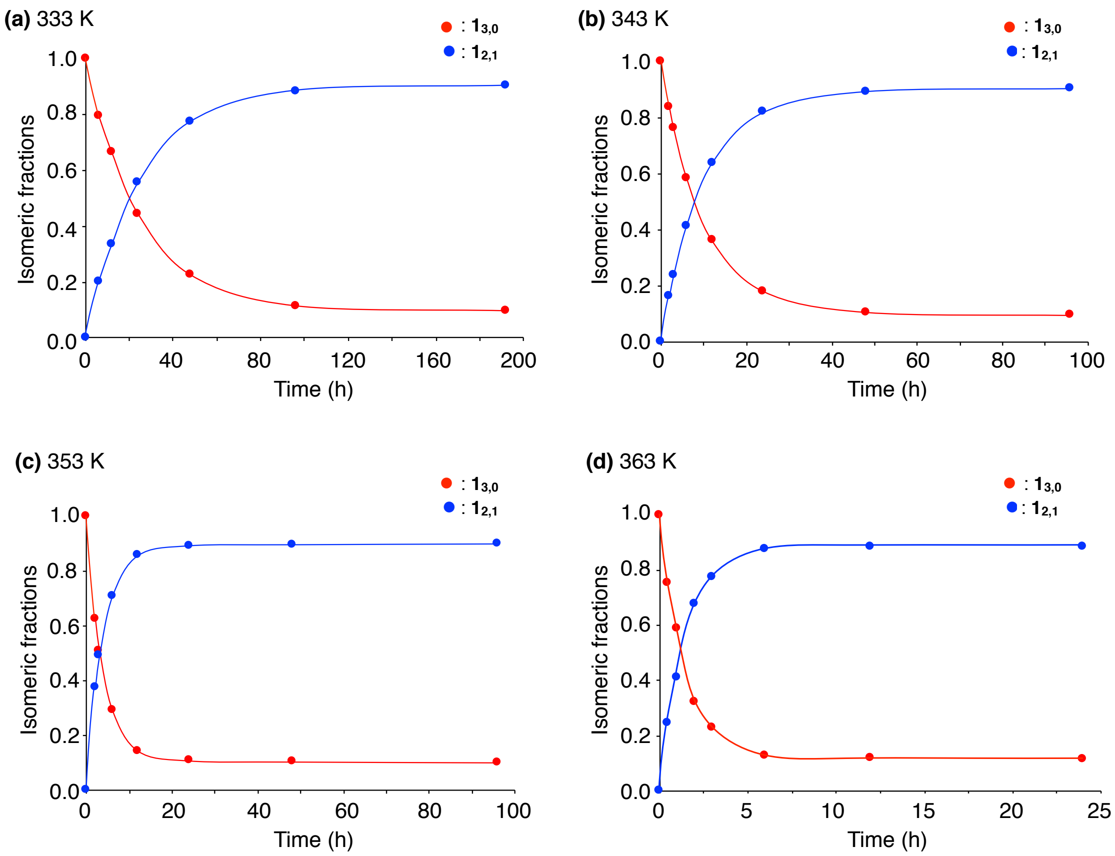

Fig. S3. Plots of the isomeric factions of $\mathbf{1_{3,0}}$ (red) and $\mathbf{1_{2,1}}$ (blue) over time obtained by the ^{19}F NMR analysis of a toluene-d_8 solution of $\mathbf{1_{3,0}}$ (initial concentration = 6.7 mM) heated at (a) 333 K, (b) 343 K, (c) 353 K and (d) 363 K. Curve fitting of the plots gave isomeric ratios at the thermal equilibrium state to be $\mathbf{1_{3,0}}$: $\mathbf{1_{2,1}}$ = 0.096 ± 0.006 : 0.904 ± 0.006 (333 K), 0.098 ± 0.002 : 0.902 ± 0.002 (343 K), 0.103 ± 0.001 : 0.898 ± 0.001 (353 K) and 0.112 ± 0.001 : 0.888 ± 0.001 (363 K). The k and k' values were calculated using the following equation; $\ln [c_{eq}/(c_t - c_{eq})] = (k + k')t - \ln K$, where c_{eq}, c_t and K refer to the equilibrium concentration of $\mathbf{1_{2,1}}$, concentration of $\mathbf{1_{2,1}}$ at t (sec) and equilibrium constant ($K = k/k'$), respectively.[7]

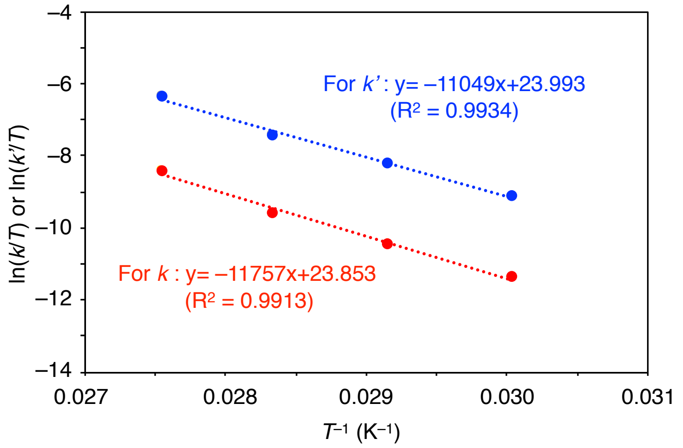

Fig. S4. Eyring plots for the thermal isomerization of **1**.

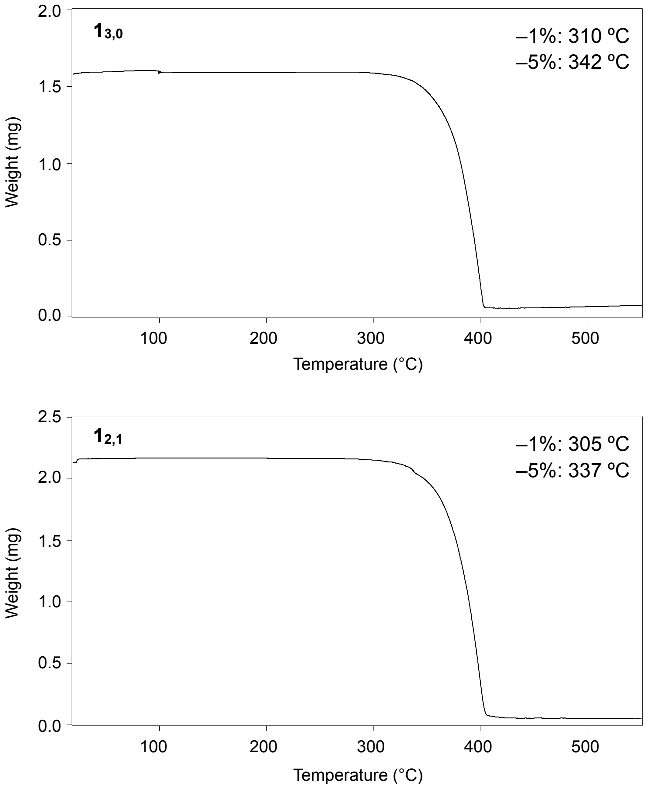

Fig. S5. TGA profiles of **1$_{3,0}$** (top) and **1$_{2,1}$** (bottom) under N_2. Heating rate = 10 °C/min.

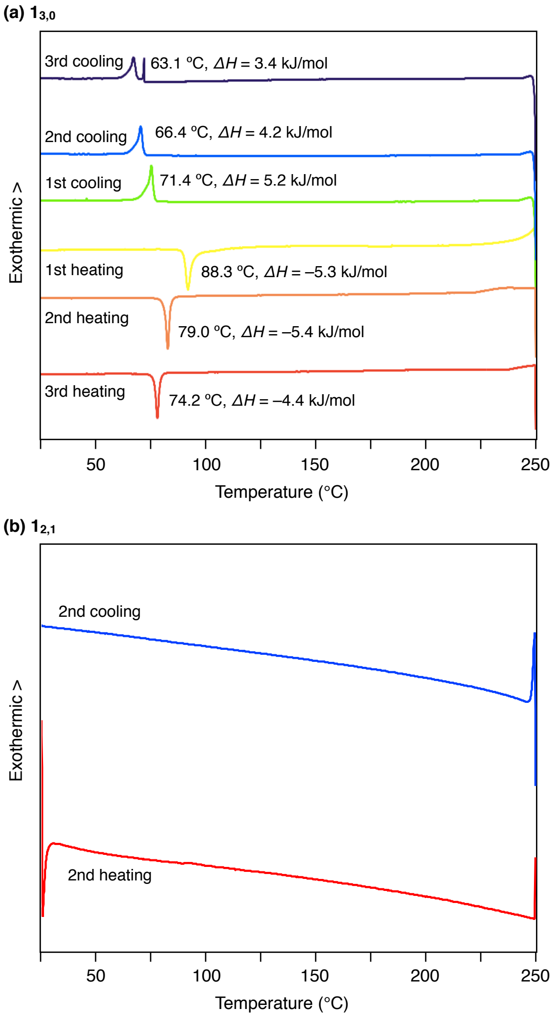

Fig. S6. DSC profiles of (a) **1**$_{3,0}$ and (b) **1**$_{2,1}$ under N_2 flow (50 mL/min) at a scan rate of 10 °C/min.

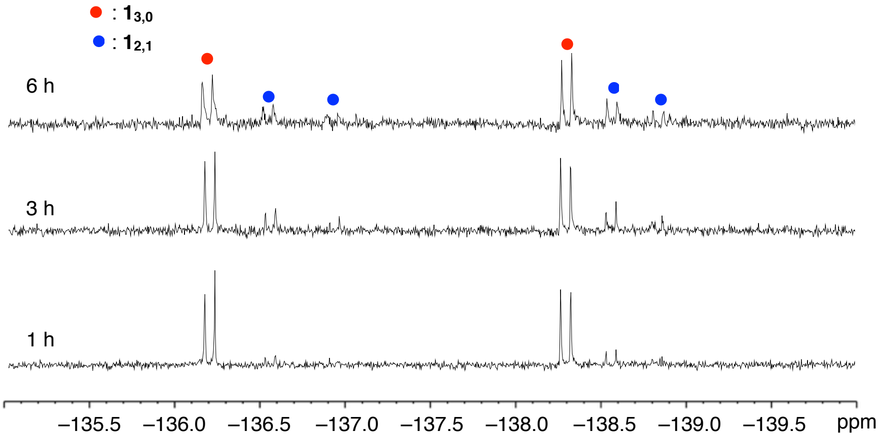

Fig. S7. ^{19}F NMR spectra (376 MHz, $CDCl_3$, 25 °C) taken after heating a crystalline sample of **1**$_{3,0}$ at 240 °C for 1, 3 and 6 h under N_2.

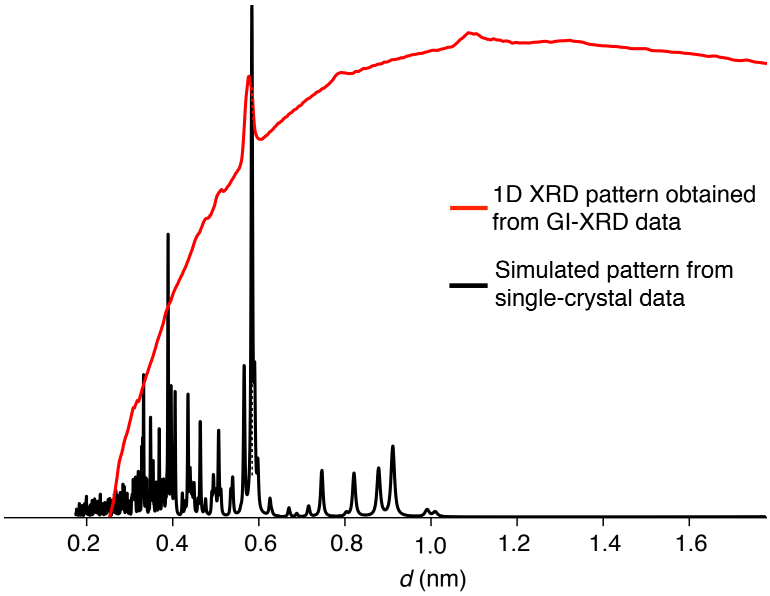

Fig. S8. Comparison of a 1D XRD profile of the thermally annealed crystalline film of $\mathbf{1}_{3,0}$ on ITO (red) obtained by the 2D GI-XRD data (Fig. 4d in the main text) and that simulated from the single-crystal X-ray structure of $\mathbf{1}_{3,0}$ (black).

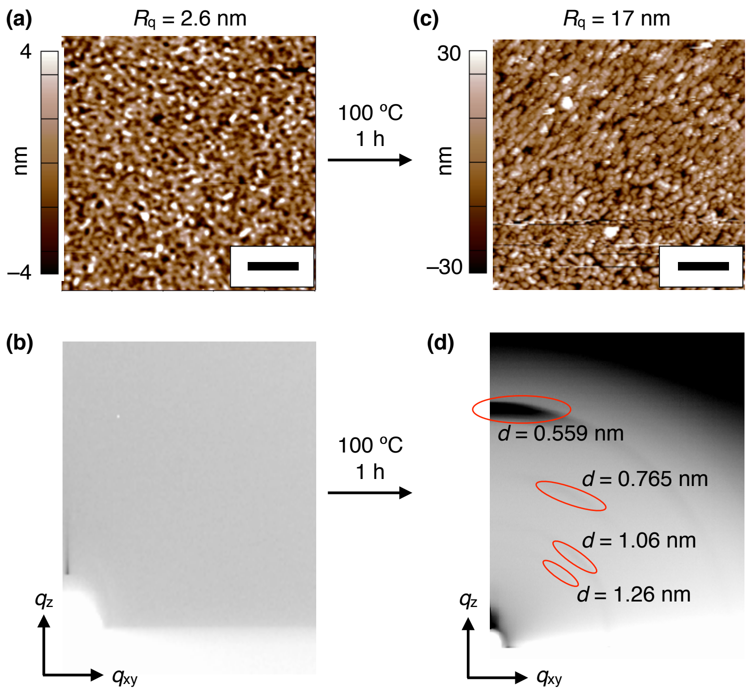

Fig. S9. (a) AFM and (b) 2D GI-XRD images of a 40 nm-thick spin-coated amorphous film of $\mathbf{1}_{2,1}$ on ITO. (c) AFM and (d) 2D GI-XRD images of a thermally crystallized film of $\mathbf{1}_{2,1}$ on ITO. Scale bars = 2 μm.

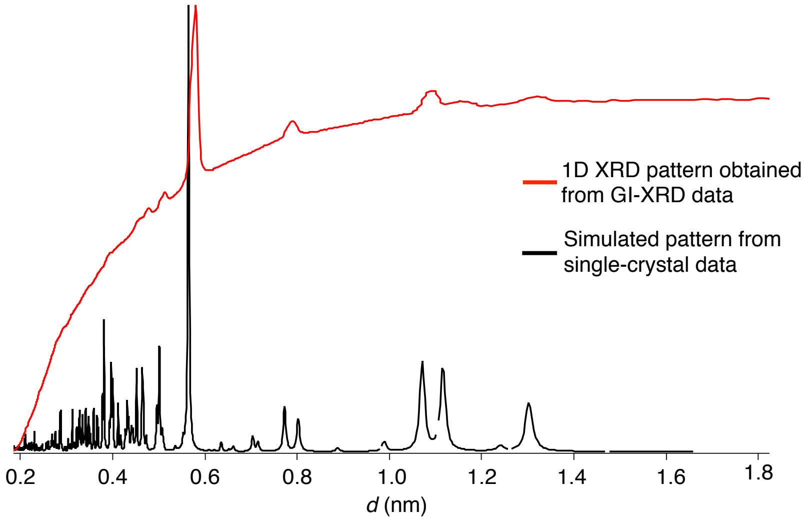

Fig. S10. Comparison of a 1D XRD profile of the thermally annealed crystalline film of $\mathbf{1}_{2,1}$ on ITO (red) obtained by the 2D GI-XRD data (Fig. S9d) and that simulated from the single-crystal X-ray structure of $\mathbf{1}_{2,1}$ (black).

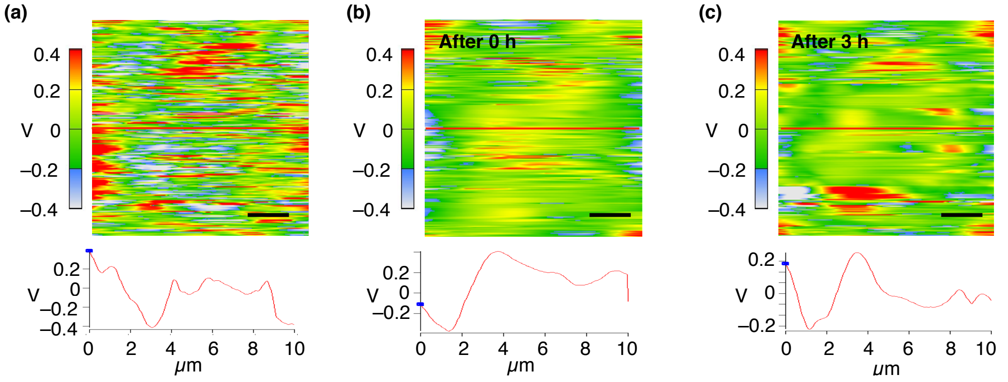

Fig. S11. (a) SKPM images (top) and surface potential trace (bottom) of a thermally crystallized film of **1**$_{3,0}$ on ITO and those measured (b) just after the application of bias voltages (±10 V) and (c) 3 h later. Bias voltages were applied according to a procedure illustrated in Fig. 5 in the main text. The surface potential traces were obtained by scanning along the red line. Scale bars = 2 μm. The corresponding AFM image of the film is shown in Fig. 4c in the main text.

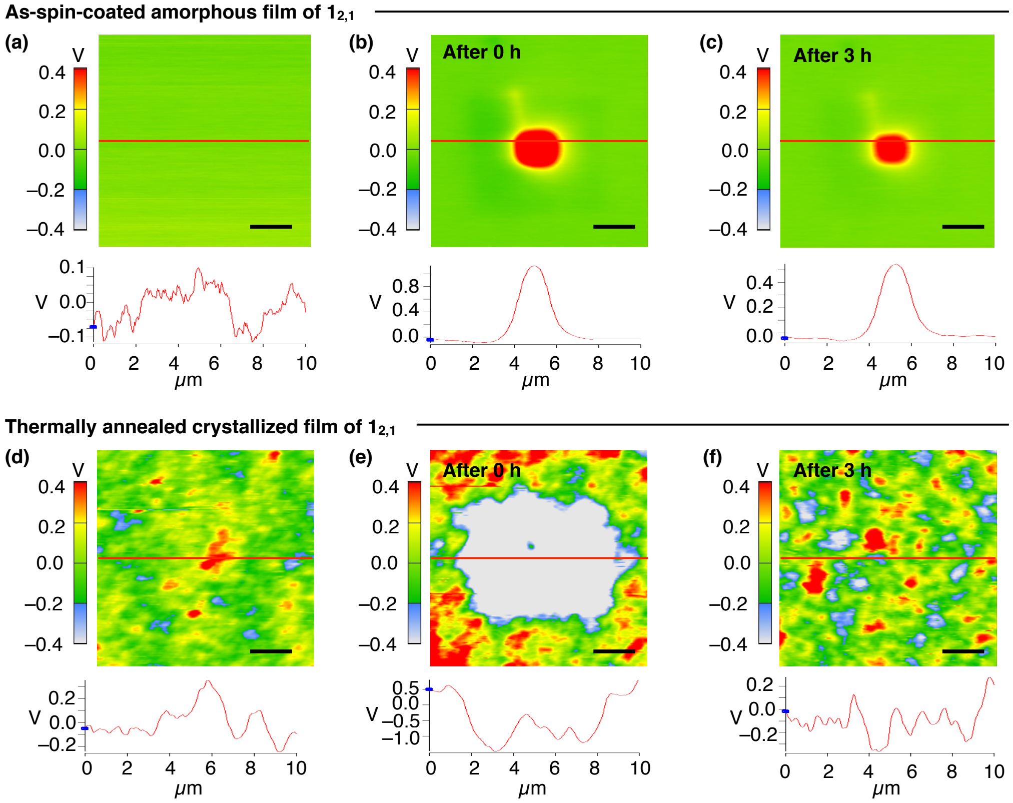

Fig. S12. (a) SKPM image (top) and surface potential trace (bottom) of a 40 nm-thick spin-coated amorphous film of **1**$_{2,1}$ on ITO and those measured (b) just after the application of bias voltages of ±10 V and (c) 3 h later. (d) SKPM image (top) and surface potential traces (bottom) of a thermally crystallized film of **1**$_{2,1}$ on ITO and those measured (e) just after the application of bias voltages of ±10 V and (f) 3 h later. Bias voltages were applied according to a procedure illustrated in Fig. 5 in the main text. The surface potential traces were obtained by scanning along the red line. Scale bars = 2 μm. The corresponding AFM images of the amorphous and crystalline films are shown in Figs. S9a and S9c, respectively.

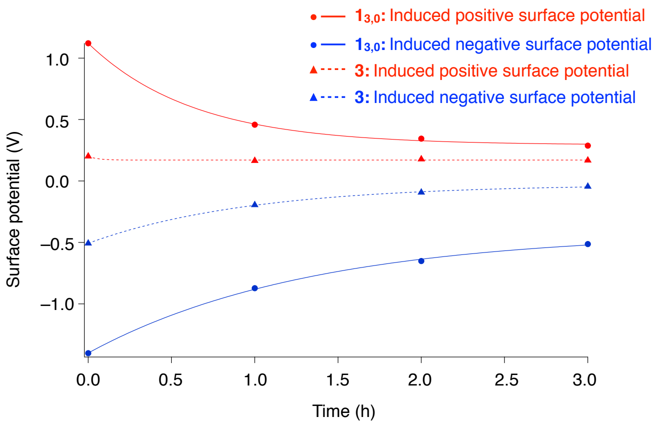

Fig. S13. Decay profiles of the induced surface potentials of 40 nm-thick amorphous films of $\mathbf{1}_{3,0}$ and **3** after the application of bias voltages of −10 V (blue) or +10 V (red).

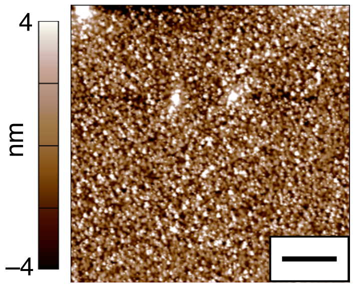

Fig. S14. AFM image of a 40 nm-thick spin-coated amorphous film of **3** on ITO. R_q = 7.0 nm. Scale bar = 2 μm.

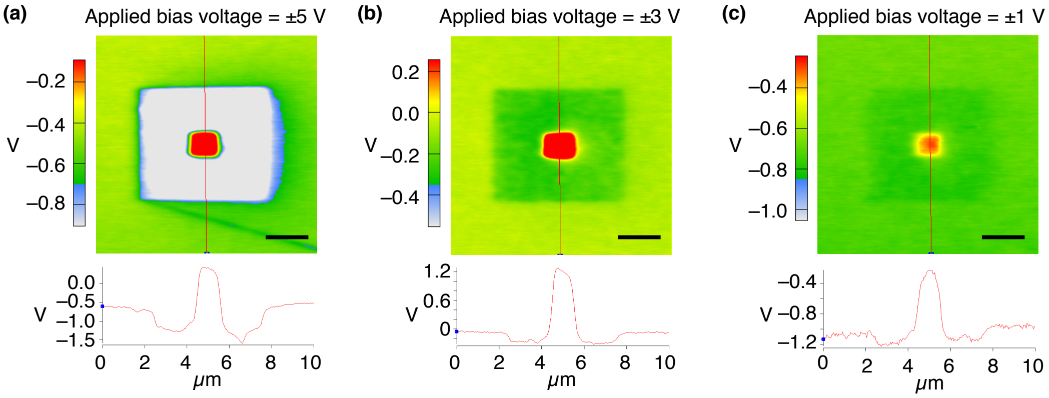

Fig. S15. SKPM images (top) and surface potential traces (bottom) of a 40 nm-thick spin-coated amorphous films of $\mathbf{1}_{3,0}$ on ITO just after the application of bias voltages of (a) ±5 V, (b) ±3 V and (c) ±1 V. Bias voltages were applied according to a procedure illustrated in Fig. 5 in the main text. The surface potential traces were obtained by scanning along the red line. Scale bars = 2 μm.

7. Supplementary References

1. L. Wang, J. Sun, Z. Huang, Q. Zheng, *CrystEngComm,* **2013**, *15*, 8511.
2. Hammersley, A. FIT2D v.17.006 (European Synchrotron Radiation Facility, 2015); http://www.esrf.eu/computing/scientific/FIT2D/
3. *SAINT,* version V7.60A, Bruker, **2009**, Bruker AXS Inc., Madison, Wisconsin, USA.
4. L. Krause, R. Herbst-Irmer, G. M. Sheldrick, D. Stalke, *J. Appl. Crystallogr.*, **2015**, *48*, 3.
5. G. M. Sheldrick, *Acta Crystallogr. Sect. A*, **2015**, *A71*, 3.
6. G. M. Sheldrick, *Acta Crystallogr. Sect. C*, **2015**, *C71*, 3.
7. N. J. Nnaji, J. U. Ani, A. M. Ekwonu, *Acta Chim. Pharm. Indica* **2013**, *3*, 212.

8. Analytical Data

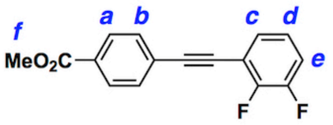

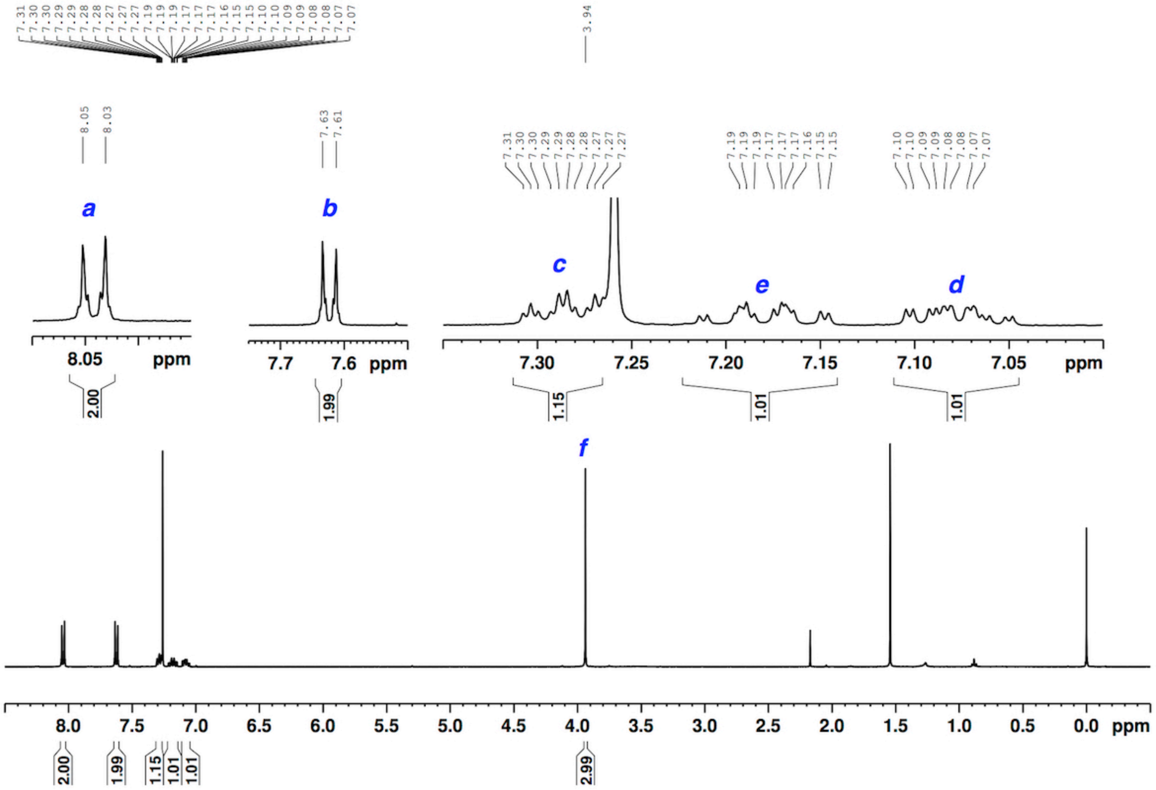

Fig. S16. ^{1}H NMR spectrum (400 MHz) of **2** in $CDCl_3$ at 25 °C.

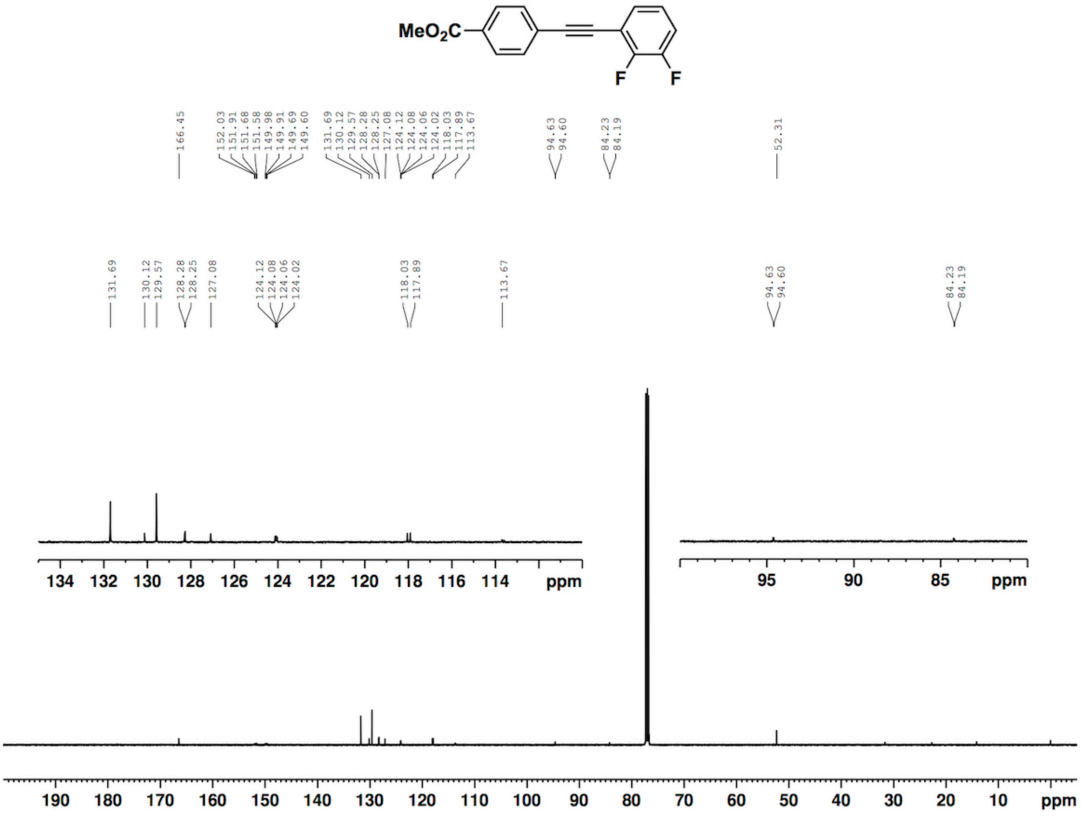

Fig. S17. ^{13}C NMR spectrum (125 MHz) of **2** in $CDCl_3$ at 25 °C.

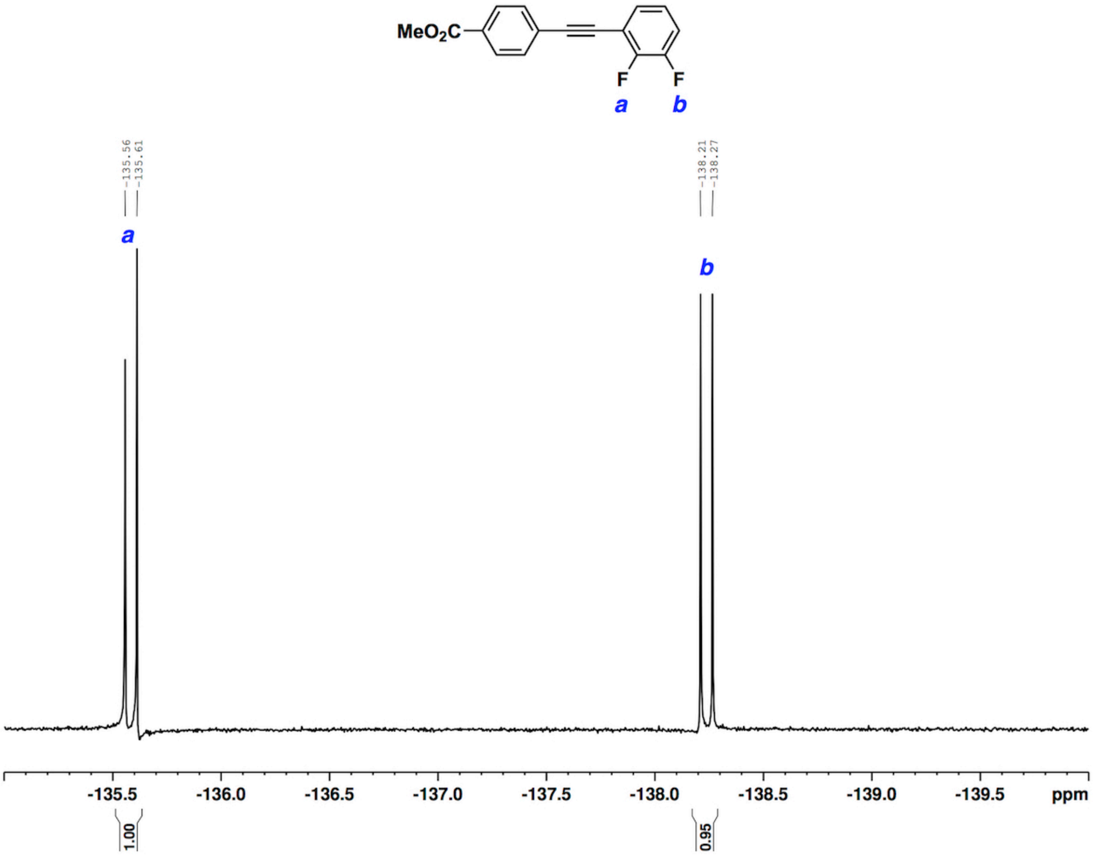

Fig. S18. ^{19}F NMR spectrum (376 MHz) of **2** in $CDCl_3$ at 25 °C.

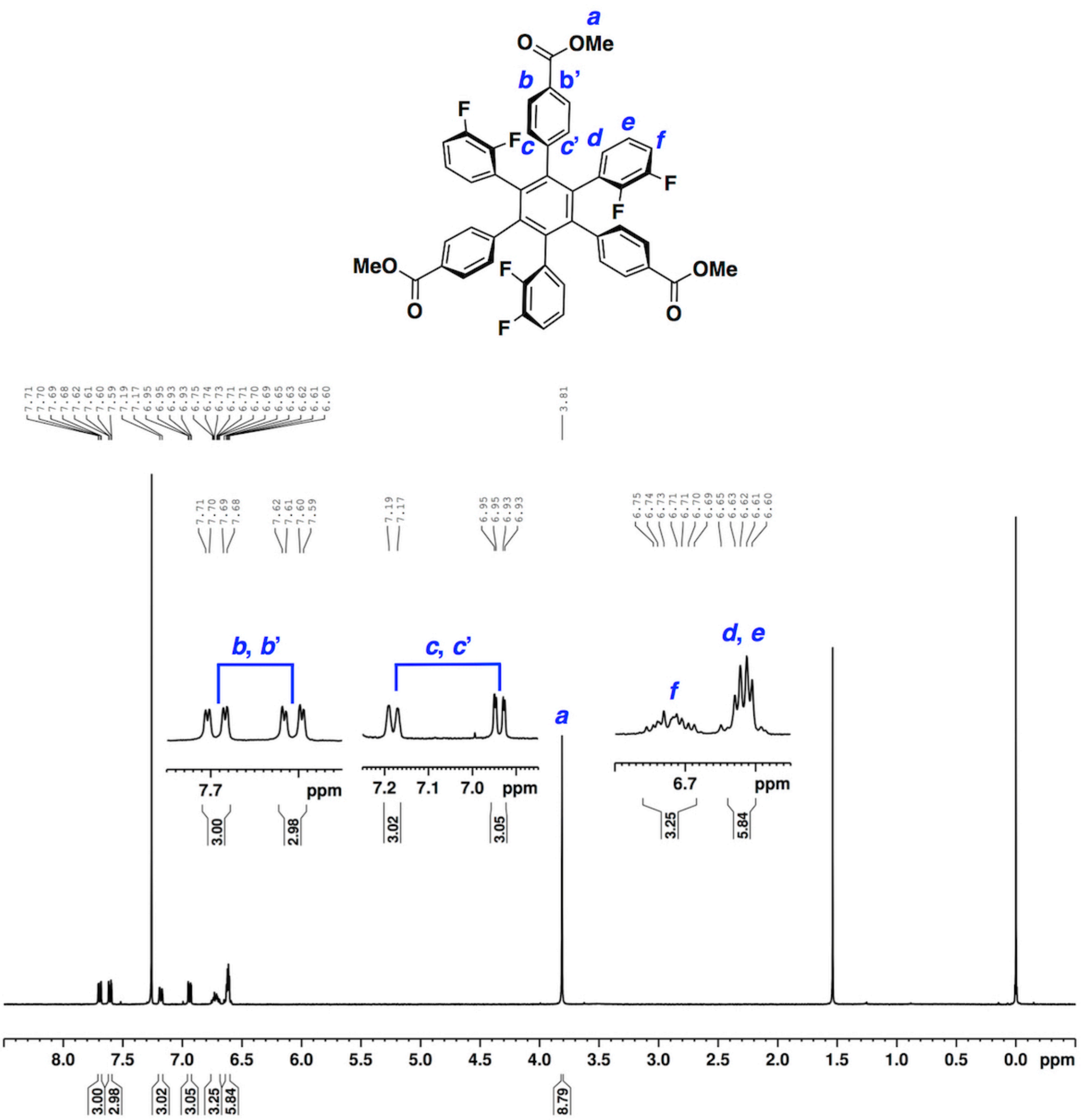

Fig. S19. ^{1}H NMR spectrum (400 MHz) of $\mathbf{1}_{3,0}$ in $CDCl_3$ at 25 °C.

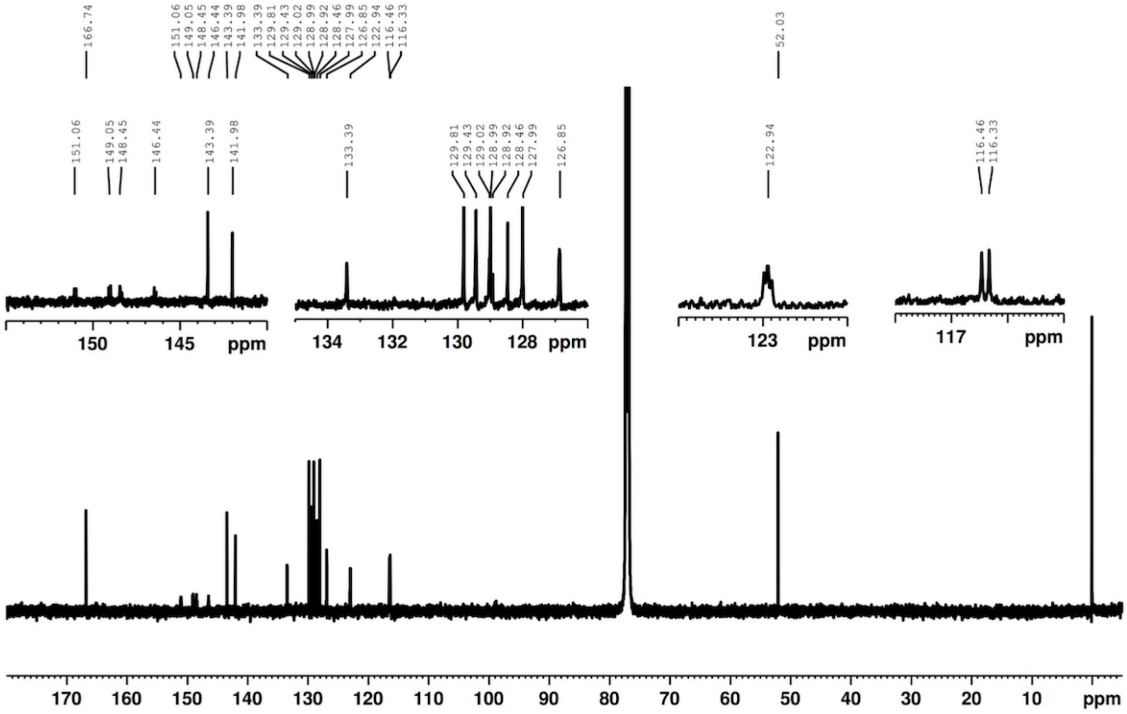

Fig. S20. ^{13}C NMR spectrum (125 MHz) of **1$_{3,0}$** in $CDCl_3$ at 25 °C.

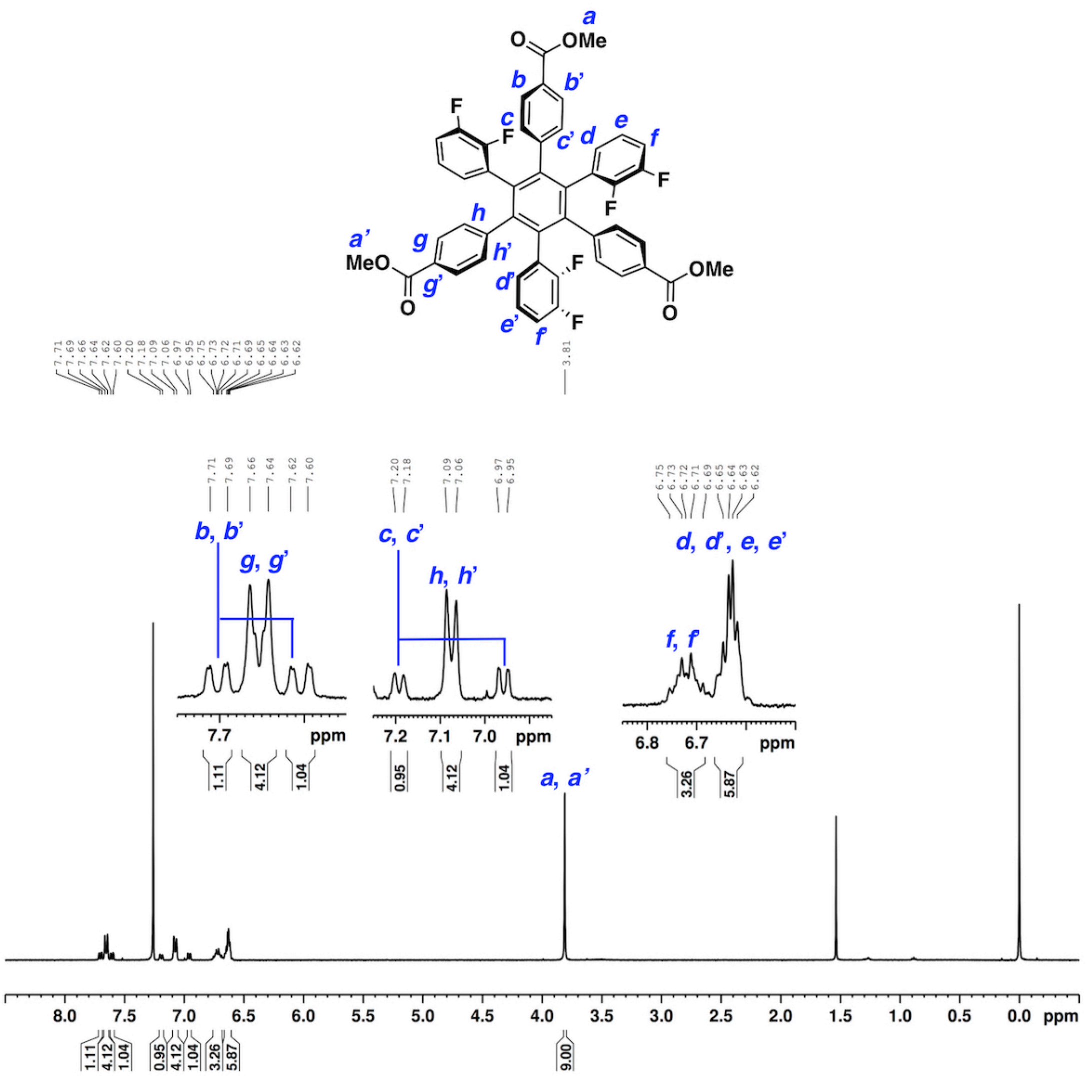

Fig. S21. ^{1}H NMR spectrum (400 MHz) of $\mathbf{1_{2,1}}$ in $CDCl_3$ at 25 °C.

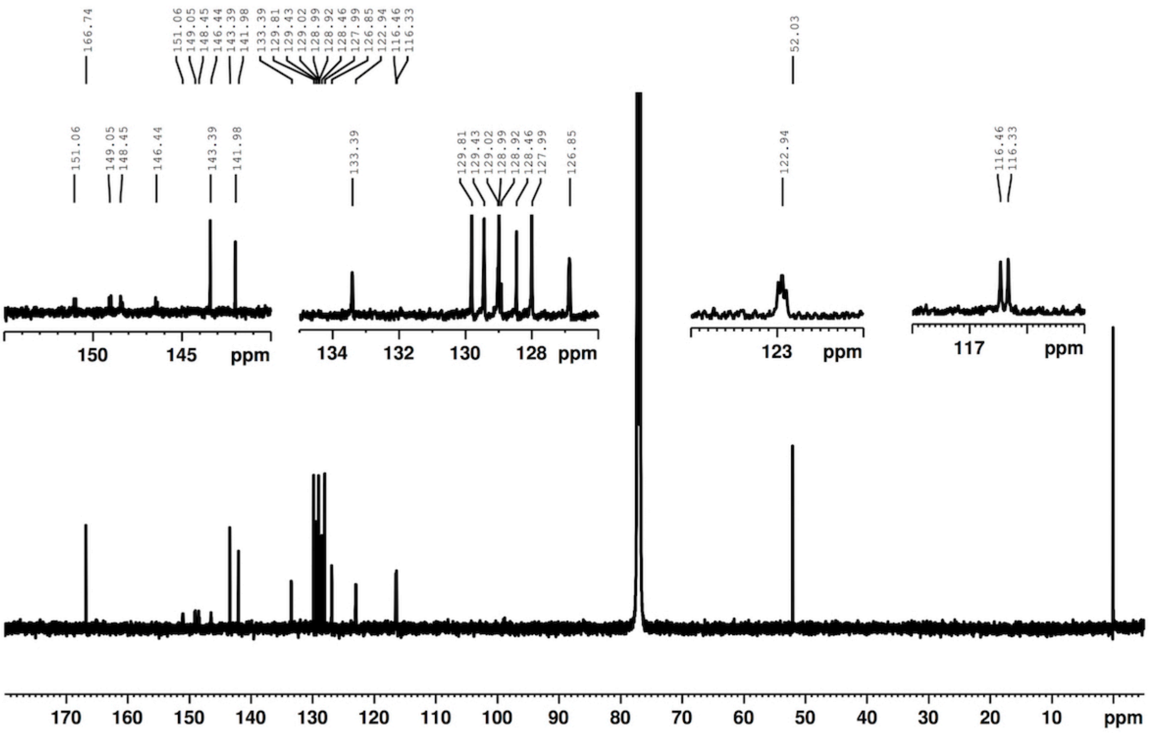

Fig. S22. ^{13}C NMR spectrum (125 MHz) of **$1_{2,1}$** in $CDCl_3$ at 25 °C.
